# A Novel In Vitro Pathological Model for Studying Neural Invasion in Non-Melanoma Skin Cancer

**DOI:** 10.3390/gels10040252

**Published:** 2024-04-08

**Authors:** Paula Ávila-Fernández, Miguel Etayo-Escanilla, David Sánchez-Porras, Cristina Blanco-Elices, Fernando Campos, Víctor Carriel, Óscar Darío García-García, Jesús Chato-Astrain

**Affiliations:** 1 Tissue Engineering Group, Department of Histology, Faculty of Medicine, University of Granada, 18016 Granada, Spain; pavila@ugr.es (P.Á.-F.); metayo@ugr.es (M.E.-E.); davidsp@ugr.es (D.S.-P.); cblanco@ugr.es (C.B.-E.); fcampos@ugr.es (F.C.); jchato@ugr.es (J.C.-A.); 2 Instituto de Investigación Biosanitaria (ibs.GRANADA), 18012 Granada, Spain; 3 Doctoral Program in Biomedicine, University of Granada, 18071 Granada, Spain

**Keywords:** advanced hydrogel models, cancer neural invasion, head and neck squamous cell carcinoma, tissue engineering

## Abstract

Neural Invasion (NI) is a key pathological feature of cancer in the colonization of distant tissues, and its underlying biological mechanisms are still scarcely known. The complex interactions between nerve and tumor cells, along with the stroma, make it difficult to reproduce this pathology in effective study models, which in turn has limited the understanding of NI pathogenesis. In this study, we have designed a three-dimensional model of NI squamous cell carcinoma combining human epidermoid carcinoma cells (hECCs) with a complete peripheral nerve segment encapsulated in a fibrine-agarose hydrogel. We recreated two vital processes of NI: a pre-invasive NI model in which hECCs were seeded on the top of the nerve-enriched stroma, and an invasive NI model in which cancer cells were immersed with the nerve in the hydrogel. Histological, histochemical and immunohistochemical analyses were performed to validate the model. Results showed that the integration of fibrin-agarose advanced hydrogel with a complete nerve structure and hECCs successfully generated an environment in which tumor cells and nerve components coexisted. Moreover, this model correctly preserved components of the neural extracellular matrix as well as allowing the proliferation and migration of cells embedded in hydrogel. All these results suggest the suitability of the model for the study of the mechanisms underlaying NI.

## 1. Introduction

Invasion and metastasis are two of the established hallmarks of cancer and a key feature of malignant cells’ dissemination from the primary tumor [1,2]. The invasion of the surrounding tissue, lymphatic spread and haematogenic spread have been considered the three most studied pathways by which solid tumors can disseminate, and much current research focuses only on these three routes of metastatic spread [3]. However, there was another route of tumor spread described back in the mid-1800s, which occurs in and along nerves, that has received little research attention. Cancer dissemination through nerves, also known as neural invasion (NI), is a key pathological feature of cancer in the colonization of distant tissues whose underlying biological mechanisms are still scarcely known [4].

NI has been studied in head and neck cancers, in which it serves as conduit for intracranial extension [2,5]. Among the different head and neck cancers, squamous cell carcinoma has reported the highest incidence of NI, as high as 80% [2,5]. NI’s intrinsic features make it difficult to diagnose and it is associated with decreased survival, increased locoregional recurrence rates, and a shorter time to recurrence [2,4,6,7]. Although NI was first described in head and neck cancers, since then many other malignancies, including pancreatic, colon and rectum, prostate, biliary tract, and stomach cancers, have reported NI features [2,8]. NI is typically associated with a poor outcome and as a harbinger of decreased survival. Recurrence rate and metastasis have been associated with positive NI and multiple studies have also demonstrated that the presence of NI leads to neurological dysfunctions such as paralysis and severe neuro-deficit [2,3,8,9].

Several NI subtypes have been described depending on which layer of the nerve stroma (epineurium, perineurium and endoneurium) is colonized by cancer cells, and nowadays the invasion of the neural tissue by a cancerous cell can be divided into seven distinct steps: (1) the survival of tumor cells, (2) the formation of a neural steady state, (3) inflammatory reaction, (4) the recruitment of tumor cells to nerves, (5) the neogenesis of nerves, (6) the adhesion of tumor cells and (7) nerve invasion [10]. However, there is controversy in the scientific community when it comes to defining the different types of NI. While several researchers define perineural invasion (PNI) as the presence of cancer cells in the perineurial space [2], others, like Liebig and collaborators, consider the presence of tumor cells in any layer of the nerve as PNI [4,10].

To date, multiple theories have been proposed to explain the pathogenesis of NI [2,3]. For the last few decades, the predominant theory of its pathogenesis involved either the need for a low-resistance rout through the nerve or the extension of lymphatic metastasis. However, the complex nerve stroma made up of overlapping collagen layers and basement membranes make access to this path anything but low-resistance and, on the other hand, several studies have demonstrated that lymphatic channels do not penetrate the inner sanctum of the nerve sheath [2,11]. NI does not represent an extension of lymphatic metastasis, nor is it merely the migration of tumor cells through a low-resistance plane [8], and evidence is emerging to indicate that the NI phenomenon is more like invasion than simple diffusion. Nevertheless, the ways in which cancer disseminates and uses nerves to invade distant tissues and the reasons why certain cancers exhibit a predilection for NI than others are as-yet poorly understood.

Our limited understanding of NI pathogenesis is a consequence of a lack of effective models able to reproduce the complex interactions between nerves, tumor cells and stroma. In this regard, tissue engineering has emerged as a powerful tool to recreate complex biological processes in a controlled and reproducible environment. Biomimetic artificial tissues are nowadays a very useful tool in disease study models [6,12,13]. However, NI’s complexity makes it difficult to build an in vitro model capable of capturing even a single aspect of the disease process. Published in vitro models are mainly limited by the difficulty in culturing peripheral nerve preparations and in replicating the neural microenvironment [2,14]. In this study, we present a hydrogel-based NI cancer model that considers the neural microenvironment and cancer cells in a reproducible in vitro system which consists of a three-dimensional model of NI squamous cell carcinoma combining epidermoid cells with a complete peripheral nerve segment immersed in a fibrine-agarose hydrogel. Fibrin-agarose advanced hydrogel has obtained successful results in a preclinical and clinical state [15,16] and also supported peripheral nerve regenerative function in vivo [17]. Now, we have used that hydrogel to build a unique model of NI that combines a highly biocompatible and natural biomaterial with the highly complex structure of the peripheral nerve to study the underlaying biological mechanism of NI. We used this fibrin-agarose gel to reproduce two vital processes of NI, a pre-invasive NI model (Pre-Inv) in which human epidermoid carcinoma cells (hECCs) were seeded on the top of the nerve-enriched stroma, and an invasive NI model (Inv) in which cancer cells were immersed with the nerve in the hydrogel.

## 2. Results and Discussion

### 2.1. Histological Analysis of Neural Invasion Models Using Hematoxylin and Eosin Staining

**Figure 1:**
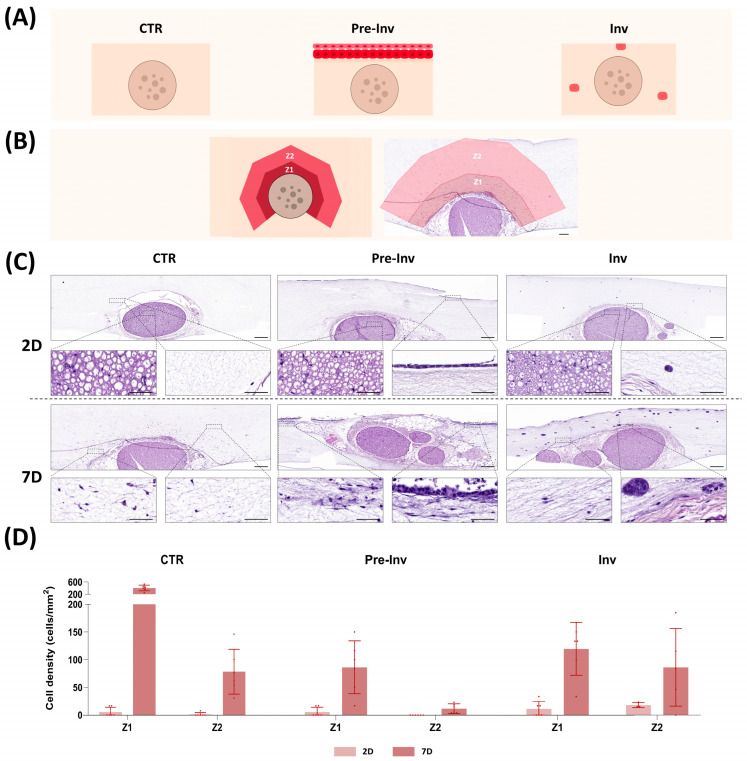
Histological evaluation of neural invasion (NI) models. (**A**) Schematic representations depicting the microscopic characteristics of each NI model. (**B**) Schematic representation of the establishment of two distinct zones, Zone 1 (Z1) and Zone 2 (Z2), within the stroma. Scale bar = 200 µm. (**C**) Histological evaluation of NI models stained with hematoxylin–eosin (HE) at different magnifications. CTR: control group model, Pre-Inv: pre-invasive model, Inv: invasive model. Scale bars of upper and bottom magnifications = 200 and 50 µm, respectively. (**D**) Quantitative analysis of cell density at Zone 1 (Z1) and Zone 2 (Z2) at each follow-up time in CTR, Pre-Inv and Inv models. Error bars represent standard deviations.

The histological analysis of the NI models using hematoxylin and eosin (HE) staining (Figure 1) revealed that the integration of a complete nerve structure and hECCs within fibrin-agarose advanced hydrogels successfully generated a microenvironment in which all elements coexisted (Figure 1C). The fibrin-agarose hydrogel integrates fibrin derived from human plasma and agarose, a natural polysaccharide that exhibits controlled self-gelling properties. Previous studies showed that the combination of these two biomaterials resulted in a significant improvement in both biological and biomechanical properties of the hydrogel and allowed the generation of a wide variety of artificial substitutes [16,17,18]. In fact, this model has already shown promising preclinical results in multiple scenarios such as skin [15], cornea [19] and oral mucosa [18] grafting and as a nerve repair strategy [17], which allowed its successful translation to clinical practice [16]. However, this is the first time this hydrogel has been used to recreate complex pathological processes such as NI.

HE staining showed that in the control group (CTR), where a complete nerve structure was embedded within the hydrogel, the infiltration of cells derived from the nerve (termed nerve-derived cells) into the gel took place after 7 days of in vitro culture. In this sense, our results confirm those obtained in several studies [20,21,22] where nerve-derived cells immersed in different hydrogels colonized and invaded artificial stroma during the initial days of culture and highlighted the trophic role of nerves in tissue development and remodeling. Interestingly, we found that the stromal layer of our model displayed two distinct zones according to the distribution pattern of the nerve-derived cells: Zone 1 (Z1), situated closest to the nerve, and Zone 2 (Z2), located further away (Figure 1B). These regions exhibited markedly different cell densities and following quantification, we found that Z1 contained higher cell density than Z2 at 7 days with a statistically significant variance in cell migration between these zones (Figure 1D) (*p* < 0.005, Supplementary Table S1).

Then, we histologically assessed the two different NI models generated by hECCs inclusion on top of the gel in a Pre-Inv NI model, or into the artificial stroma in an Inv NI model.

First, we found that the Pre-Inv NI model allowed hECC seeding in the top of the hydrogel while preserving the complex nerve structure throughout the study period. In addition, HE staining revealed a progressive development of the squamous cell carcinoma epithelium in the Pre-Inv model (Figure 1C) exhibiting increased epithelium thickness and maturity after 7 days, as evidenced by the epithelial desquamation process. Such differentiation features were in concordance with other published studies that successfully demonstrated the suitability of tissue-engineered pathological models of squamous cell carcinoma to recreate the morphological appearance and histological characteristics of tumor cell invasion [23]. At the same time, in the stroma, nerve-derived cells infiltrated and colonized the artificial dermis, as in the CTR group. However, the quantification of nerve-derived cells revealed a significant reduction in their invasion ability compared to the CTR group in both zones at 7 days of in vitro culture (Figure 1D) (*p* < 0.005 in both cases, Supplementary Table S1).

In addition, we found that in the Inv model, the incorporation of hECCs into the stroma showed typical cancer pathological features such as the formation of cell aggregates, as shown in Figure 1C. These results are in line with published studies that demonstrated the ability of biomimetic hydrogel to support the initiation and growth of tumor organoids [24]. Notably, these cancerous aggregates appeared to be in close proximity to the outer layer of the epineurium of the nerve explant after 7 days. Similar to the Pre-Inv model, a quantitative analysis indicated significant differences in cell density as compared to the CTR group (Figure 1D). In this model, the presence of hECCs in the dermis significantly influenced the migration and colonization of nerve-derived cells, leading to a notable reduction in cell density in Z1 compared to the CTR group (*p* < 0.005, Supplementary Table S1).

The histological evaluation shown here supports the idea that fibrin-agarose hydrogel allows the generation of an advanced NI model that combines structures with high architectural complexity, such as nerves with tumor cells. This combination was able to mimic the multifaceted scenario in an artificial stroma and could favor the establishment of molecular crosstalk and chemiotaxis communication between the different players of the NI process as they mimic the pathological process of squamous cell carcinoma.

However, our Pre-Inv and Inv ex vivo NI models showed very different features to other published NI study models. Most published ex vivo NI models cocultured cancer cells with nerve explants in an inaccurate physical scenario. To date, most NI models have been based on 2D culture plates [25], the use of direct cancer nerve interactions [26] or the use of organoids [27], lacking the critical interaction with the stroma which is in turn, essential for the interplay among the various key players involved in NI processes [14,26,27,28]. Interestingly the unique design of our NI models highlighted the complex crosstalk between nerve and cancer cells evidenced by the impact on nerve-derived cells’ migration ability. The incorporation of cancer cells in both the epithelium and the stroma influenced the migration capacity of nerve-derived cells. These results corroborate the diffusion properties of the hydrogel used. However, to properly assess the effect of incorporating hECCs on the migration of nerve-derived cells in Pre-Inv and Inv models, more studies are still needed.

### 2.2. Histochemical Assessment of Extracellular Matrix Components in Neural Invasion Models

**Figure 2:**
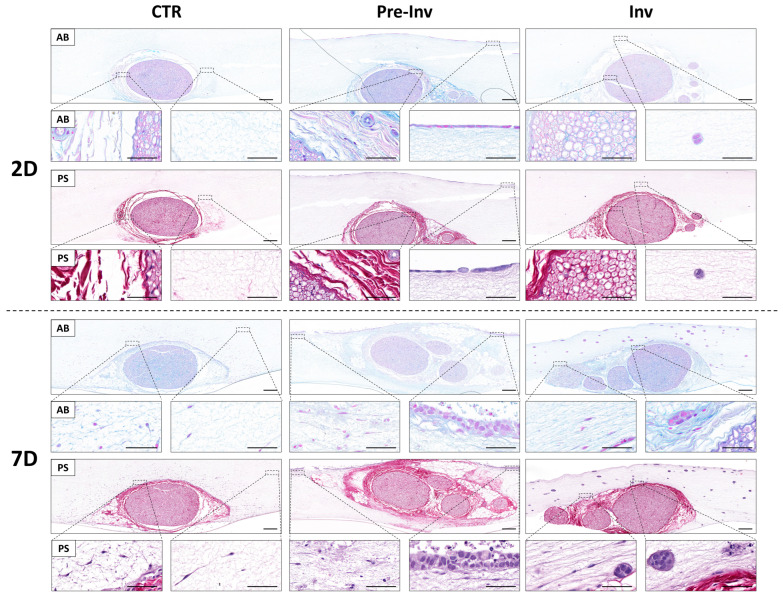
Assessment of extracellular matrix components in neural invasion models. Histochemical signals for alcian blue (AB) and picrosirius red (PS) staining. Images are shown at various time points (2 and 7 days) in each tissue sample. CTR: control group model, Pre-Inv: pre-invasive model, Inv: invasive model. Scale bars of upper and bottom magnifications = 200 and 50 µm, respectively.

Given the significant influence of the complex extracellular matrix (ECM) of the nerve stroma on mimicking NI, we assessed two crucial ECM components using histochemical staining (Figure 2). We employed alcian blue histochemical staining (AB) for proteoglycan evaluation and picrosirius red staining (PS) to examine collagen fibers (Figure 2).

The CTR group revealed an adequate maintenance of the typical nerve stroma, primarily composed of overlapping collagen fibers, throughout our studied time (Figure 2).

We further examined the potential emergence of newly formed ECM components by nerve-derived cells infiltrating the artificial stroma, yet no such expression was observed after 7 days of ex vivo culture in the CTR group (Figure 2). This evidence was also found in both the Pre-Inv and Inv models of NI, which similarly exhibited preserved stromal nerve explants and integrity after 7 days of culture, with no evident signs of de novo ECM formation or destruction by cancer cells (Figure 2).

ECM molecules are synthesized and deposited by cells over time as they interact with their environment [29]. In vitro models typically require more time for cells to express and assemble ECM components, mimicking the natural process that occurs in vivo. The results shown in our NI models are in agreement with previously published studies, which demonstrated that more in vitro culture time is needed for ECM molecule production [15].

On the other hand, the ECM serves as a dynamic scaffold that not only provides structural support but also plays a pivotal role in regulating various cellular processes such as adhesion, migration and signaling [29]. In the context of NI, alterations in the ECM composition and organization can significantly influence the invasive behavior of cancer cells towards nerve structures [10]. Actually, ECM protein composition is one of the most important regulators of NI process [30]. The interaction between cancer cells and nerve stroma is complex and multifaceted, involving reciprocal signaling pathways and crosstalk between the tumor microenvironment components. In this study, we have not detected any serious disturbance in the main nerve components of the ECM analyzed. These findings align with the existing literature, which indicates that changes in the ECM require extended periods to manifest detectable alterations via histochemical analysis [31,32]. However, longer time points could reveal ECM alterations, as shown in a recently published study [33] which demonstrated that ECM stiffness is associated with NI in vivo, and that NI in vitro is induced with stiffener hydrogels. In this study, the question of whether cancer cells are influencing native nerve stroma should be further studied with longer time points and specific protein detection analysis and molecular expression profiles.

### 2.3. Analysis of Cell Proliferation in Neural Invasion Models

**Figure 3:**
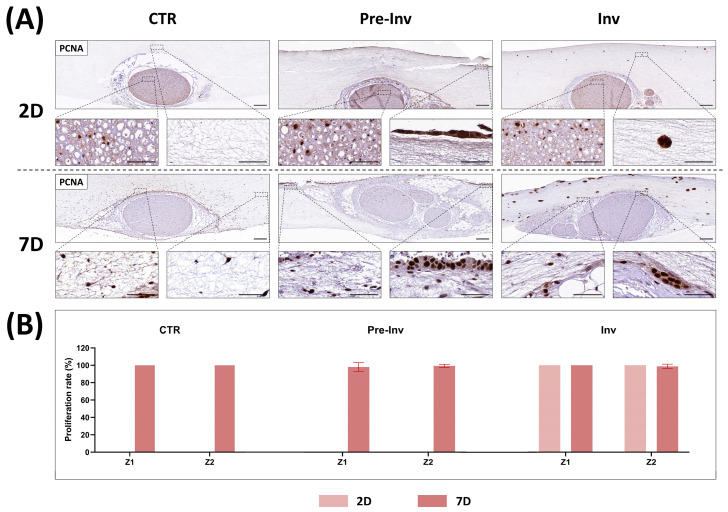
Analysis of cell proliferation in neural invasion models. (**A**) Immunohistochemical detection for PCNA at different time points (2 and 7 days) in each experimental group. CTR: control group model, Pre-Inv: pre-invasive model, Inv: invasive model. Scale bar of upper and bottom magnifications = 200 and 50 µm, respectively. (**B**) Quantitative analysis results of the proliferative rates expressed as the percentage of positively stained cells. Error bars represent standard deviations.

To determine the dynamics of cell proliferation in our NI models, we assessed the PCNA proliferative marker (Figure 3). The immunohistochemical detection of PCNA showed that some cells within the nerve explant at 2 days exhibited positivity for PCNA in the intrafascicular layer of the CTR group. Notably, at this time point no cells were observed in the stroma layer of the model, nor in Z1 or Z2 (Figure 3A). However, cells originating from the nerve and invading the stroma at 7 days exhibited high rates of PCNA positivity. Quantification revealed that all nerve-derived cells colonizing the artificial stroma displayed PCNA positivity, with a proliferative rate of 100% observed in both Z1 and Z2 (Figure 3B).

In the Pre-Inv and Inv NI models, PCNA analysis revealed the active proliferation of cancer cells incorporated into the hydrogel at both 2 and 7 days of culture. In the Pre-Inv model, hECCs formed a highly proliferative epithelium even after just 2 days of culture. Additionally, nerve-derived cells colonizing the stroma also exhibited high rates of proliferation (Figure 3B). In the Inv model, hECCs were embedded within the stroma and maintained proliferative activity from day 2 onwards. At 2 days, cell aggregates consisted of 1–2 cells and progressed to larger aggregates containing 5–7 cells, all displaying high proliferative activity. Notably, proliferatively active cell aggregates were also observed in physical contact with the epineurium barrier of the nerve segment (Figure 3A). These findings were further supported by cell quantification in Figure 3B, where after 7 days of culture, the proliferation rate remained consistently around 100% for both Z1 and Z2.

The ability of cancer cells not to depend on exogenous stimulus for growth and proliferation is known as “self-sufficiency in growth signals”, and it is one of the main characteristics of tumor cells [1]. The results of our NI models demonstrated an actively proliferative state of hECCs in the Pre-Inv and Inv model and confirmed cancer features in all studied times (Figure 3A). In addition, the Inv NI model showed the physical interaction between cancer cell aggregates and nerve stroma structures while maintaining the intrinsic proliferative capacity of the tumor cells, as shown in Figure 3A. These results are in line with other NI studies that demonstrated an active expression of proliferative markers in NI’s surrounding areas [34].

Additionally, the high rate of proliferation expression of PCNA in nerve-derived cells reflects their dynamic response to both intrinsic and extrinsic factors within the neural microenvironment [22,35]. Nerve-derived cells, such as Schwann cells and fibroblasts, possess inherent regenerative capabilities, requiring active proliferation in response to injury or pathological stimuli. Such intrinsic capability is evidenced by the PCNA positivity found in the intrafascicular compartments of the nerve explant at 2 days in all our NI models. In addition, the presence of growth factors and signaling molecules within the neural microenvironment can also influence the proliferation capability of nerve-derived cells. The ECM composition of nerves, characterized by specific matrix molecules and cell adhesion proteins, can facilitate cell proliferation by providing structural support and signaling cues. Here, all nerve-derived cells exhibited high proliferation rates, which validated the model’s capability to simulate the behavior of a biologically active cancer, which is in turn characterized by a high proliferation index. These results are consistent with previously published ex vivo models of NI that utilized Matrigel invasion assays to induce pancreatic carcinoma invasion into the nerves [26]. This study also demonstrated a high viability and proliferation of cancer cells, as well as fibroblast-like cells, along with increased cell growth of cancer cells distributed as aggregates, as observed in our Inv model. However, further studies should determine the exact mechanisms underlaying cell-derived nerve proliferation and differentiation.

### 2.4. Phenotypic Characterization of Nerve-Derived Cells in Neural Invasion Models

**Figure 4:**
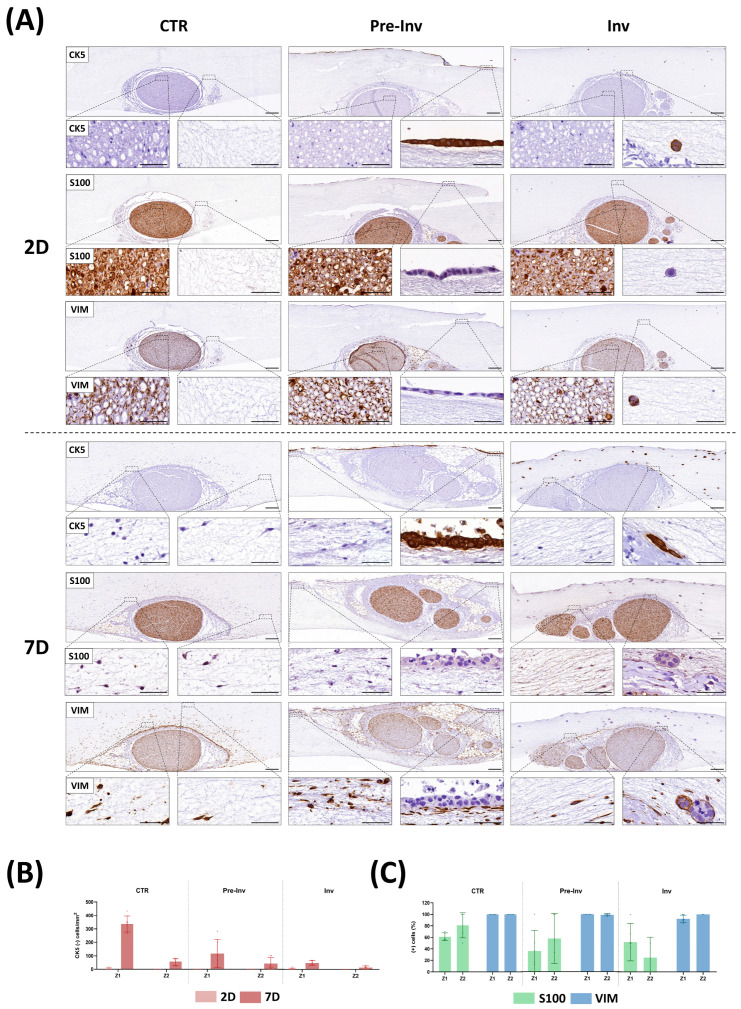
Histochemical assessment of cellular profile in neural invasion models. (**A**) Immunohistochemical signal for CK5, S100 and VIM at various time points (2 and 7 days) in each tissue sample. (**B**) Quantitative analysis results of the number of cells migrating from the nerve per mm^2^ at 2 and 7 days of culture. (**C**) Quantitative analysis results of S100-positive cells and VIM-positive cells at 7 days of culture. CTR: control group model, Pre-Inv: pre-invasive model, Inv: invasive model. Scale bars of upper and bottom magnifications = 200 and 50 µm, respectively. Error bars represent standard deviations.

To investigate the influence of hECC inclusion on nerve-derived cell invasion, we conducted various immunohistochemical detections to distinguish between different cell populations, as illustrated in Figure 4.

Firstly, we used the epithelial-specific CK5 marker to assess the phenotype of hECCs in our NI models. The CTR group exhibited no CK5 expression at any of the studied time points, neither in the nerve explants nor in the nerve-derived cells colonizing the stroma, as hECCs were not included in this model. Conversely, in the Pre-Inv and Inv models, only hECCs were readily identified with the CK5 marker, confirming the epithelial nature of this non-melanoma skin cancer. This differential CK5 expression enabled effective discrimination between the included hECCs and the nerve-derived cells that invaded the stroma. Furthermore, this marker facilitated the determination of cell density in different areas, considering only the cells originating from the nerve, by their negative expression for CK5 (Figure 4B). The assessment of nerve-derived cell invasion using the CK5-negative marker revealed intriguing changes in the different NI models. In the CTR group, there was a noticeable increase in cells invading the artificial stroma at 7 days, particularly in Z1, as compared with 2 days (Figure 4B). Interestingly, this immunostaining clearly illustrated the impact of hECC inclusion in the Pre-Inv and Inv models on the cell invasion ability. In the Pre-Inv model, a statistically significant reduction in CK5-negative cells in Z1 was observed at 7 days of culture compared to the CTR group (*p* < 0.005, Supplementary Table S1). This reduction was even more pronounced in the Inv NI model. Here, hECCs included in the stroma significantly reduced cell migration from the nerve at 7 days in both stromal zones (Z1 and Z2) (*p* < 0.005 for Z1 and *p* < 0.05 for Z2, Supplementary Table S1). These results align with the previous analysis and confirm the impact of cancer cells on the migration of nerve-derived cells.

The evidenced influence of hECC inclusion in our NI models suggests a multifaceted interplay between cancer cells, stromal elements and the tumor microenvironment. These findings also corroborate previously discussed histological results, indicating a paracrine influence of cancer cells on normal nerve-derived cells. Our NI models facilitated molecule diffusion, fostering continuous crosstalk between cancer and stromal cells. This dynamic interaction underscores the complexity of tumor progression, wherein cells navigate through intricate microenvironments and adapt their migration strategies [36]. The organization of the tumor microenvironment, particularly the stromal architecture, plays a pivotal role in dictating the mode and dynamics of invasion [37]. Future studies aimed at elucidating the combined effects of stromal network structure, tumor cell signaling, and extracellular signaling cues on cancer cell migration and metastasis, providing further insights into the mechanisms driving metastatic dissemination.

On the other hand, several studies have emphasized the crucial role played by Schwann cells in facilitating tumor cell invasion into nerves and orchestrating the preliminary stages leading to NI [10,38,39]. During early NI, Schwann cells exhibit migratory behavior towards tumor cells, disrupting tumor cell aggregates and fostering tumor invasiveness to guide tumor cells towards nerve structures [10,38,39]. Given these crucial roles, immunodetection utilizing S100 and vimentin (VIM) markers were employed to delineate the phenotype expression profile of nerve cells infiltrating the stroma in NI models, as depicted in Figure 4A. The intrafascicular examination of these markers in the CTR group affirmed that this advanced hydrogel maintained the normal expression of neural S100 and stromal VIM cell markers over time in the nerve explant (Figure 4A). Interestingly, when assessing the invasion ability of the nerve-derived cells in the artificial stroma of the CTR group, we observed positive cells for both markers after only 7 days of culture. Cell quantification, as depicted in Figure 4C, revealed that all cells invading the artificial stroma were VIM-positive cells in both studied zones (Z1 and Z2), with only 61% being S100-positive in Z1 and 80% in Z2. Remarkably, in both the Pre-Inv and Inv NI models, not only did the cell density change, but the cell’s phenotypic profile also altered after hECC inclusion (Figure 4A,C). Specifically, a reduction in S100-positive cells was observed in both studied zones (Z1 and Z2) in the Pre-Inv and Inv models compared to the CTR group, although no statistically significant differences were found (*p* > 0.05, Supplementary Table S1). Curiously, published investigations using NI models revealed that Schwann cells are actually highly cancer-affine cells able to migrate toward cancer cells before these even start an invasion [28]. This indicates that cancer cells can modify the behavior of Schwann cells, as also observed in our study. Although our study only showed minor non-statistical variations in this aspect, future research with longer time points should delve into the dynamic alterations in this model more comprehensively.

## 3. Conclusions

In conclusion, NI is a complex process that involves bidirectional communication between the tumor and peripheral nerve (nervous tissue and stromal cells), with multiple factors contributing to its progression. While much remains unknown about NI, recent research has focused on the neural niche/microenvironment as a key organizer. However, studying NI accurately has been challenging due to the limitations of two-dimensional models and the inability of current three-dimensional models to fully mimic the neural microenvironment. In our study, we propose advanced fibrin-agarose hydrogel-based models that combine tumor cells with a complete peripheral nerve segment in an artificial microenvironment to elucidate the underlying mechanisms of NI. Our results demonstrated that these NI models allowed for the combination and maintenance of nerve and cancer cells’ coexistence, exhibiting proliferation and migration capacity. Furthermore, our Pre-Inv and Inv models replicated the neural niche and demonstrated crosstalk between nerve and cancer cells with an evident impact on cell migration. This model has considerable relevance in the fields of translational and precision medicine as it could serve as a platform for studying patient-specific responses to NI, identifying biomarkers linked to aggressive invasion phenotypes and guiding tailored treatment plans. Additionally, it enables efficient drug screening and supports the development of targeted therapies tailored to address neural invasion mechanisms effectively. However, our study has certain limitations that warrant consideration, such as the limited duration of culture time points, the necessity for comprehensive molecular analysis, including protein and gene expression assessments, and the necessity for integrating additional cell types. Moving forward, future research investigations will focus on overcoming these limitations by conducting longer-term studies to capture dynamic changes over time, performing a comprehensive molecular characterization and validating drug tests for novel anti-tumoral treatments in vitro.

## 4. Materials and Methods

### 4.1. Cell Sources and Sample Acquisition

The generation of different models of NI has involved the use of different cell lines. First, the standardized human epidermoid carcinoma cell (hECC) line A431, obtained from the American Type Culture Collection (ATCC, CRL-155), was used as epithelial cell source. These cells were cultured in Dulbecco’s Modified Eagle’s Medium (DMEM, Sigma-Aldrich, D6429, St. Louis, MO, USA) supplemented with 10% Fetal Bovine Serum (FBS, Sigma-Aldrich, F7524) and 1% antibiotic/antimycotic solution and cultured under standard conditions at 37 °C in a humified incubator (Esco Lifesciences Group, Changi, Singapore) with 5% CO_2_. Once cells reached 70% confluence, they were trypsinized with 0.25% trypsin-ethylenediaminetetraacetic acid (EDTA) solution (Sigma-Aldrich, T3924).

The day before the generation of NI models, rat sciatic nerves from 12-week-old Wistar rats weighing approximately between 203 and 280 g (Janvier Labs, Le Genest-Saint-Isle, France, authorization number D53-103-02) were harvested at the Animal Facilities of the University Hospital Virgen de las Nieves, Granada, Spain, following standardized protocols [17,40]. Nerves were preserved in phosphate-buffered saline solution (PBS) supplemented with 2% antibiotic/antimycotic solution for 3 h. Subsequently, adjacent connective tissue was carefully removed, and nerves were dissected into fragments of 5 mm. Then, the segments of sciatic nerves were preserved at 4 °C in 1% antibiotic/antimycotic PBS overnight.

All animal procedures were conducted according to the Spanish and European regulations for animal experimentation (EU directive No. 63/2010, RD 53/2013) and approved by the Ethics and Animal Experimentation Committee of Granada University, approval No. 29-03-2022-052 (grant No. FIS P20-0318).

### 4.2. Generation of Neural Invasion Models

In this study, we followed previously described protocols [15,41] to generate fibrin-agarose-based models for investigating the role of different cell sources in the dissemination and NI of hECCs (Figure 1A). Briefly, 380 µL of human plasma was mixed with 62.5 µL of DMEM, 7.5 µL of tranexamic acid (Amchafibrin, MEDA Pharma, Madrid, Spain), 25 µL of 2% CaCl_2_ and 25 µL of 2% type VII agarose in PBS, resulting in stromal substitutes with a volume of 0.5 mL. This mixture was then poured into 12-well porous inserts (83.3931.040, Sarstedt, Nümbrecht, Germany) until complete gelation occurred, with nerve fragments pre-positioned within the inserts.

Three distinct models were created by combining fibrin, obtained from human plasma donors, with 2% type VII agarose (A9045, Merck, Darmstadt, Germany). The CTR model consisted of adding this acellular mixture to the inserts. In the Pre-Inv model, following gelation, 500 µL of DMEM containing 1,000,000 cells/mL of hECCs was added to the top surface. Lastly, the Inv model was generated by adding 1,000,000 cells/mL of hECCs to the hydrogel mixture containing 62.5 µL of DMEM.

The study models were cultured under standard conditions (37 °C and 5% CO_2_) and samples were collected for analysis after 2 and 7 days of follow-up.

### 4.3. Histological Characterization of Neural Invasion Models

To gain a deeper understanding of cell–cell interactions and the involvement of cancer cells in neural spread and invasion, a histological characterization was conducted, as previously described [40,42,43].

Initially, the study models were fixed in neutral buffered 4% formaldehyde (Panreac, 252931.1214) for 24 h at room temperature. Subsequently, they were dehydrated, cleared, embedded in paraffin, and sectioned into 5 µm slices. Tissue sections underwent histological analyses to examine the cellular organization and morphology of various cell types and tissue structures within the artificial models. An HE stains technique was employed for this purpose.

Furthermore, the presence of fibrillar collagens and proteoglycans was assessed using PS and AB histochemical methods, respectively. These additional analyses were used to study the composition and characteristics of the ECM components present in the study models. These histochemical analysis were conducted following standardized protocols [44,45,46,47].

To evaluate the cellular behavior of our models, an immunohistochemical analysis of the proliferation cellular nuclear antigen marker (PCNA) was performed [48]. In addition, to evaluate the distribution pattern of the different cell types in the study models, immunodetection with the CK5 (epithelial cells) [49], S100 (Schwann cells) [50] and VIM [51] markers allowed us to determine the expression profile of the different cells leaving the nerve. The technical details of the immunohistochemical procedures and antibodies used are summarized in Table 1.

### 4.4. Statistical Analyses

Quantitative histological analyses were performed using the ImageJ software (version 1.53k, National Institute of Health, Bethesda, MD, USA), as detailed in previous studies [40,52,53]. First, in the different artificial models we identified two different cellular enriched zones, Zone 1 (Z1), which consists of a 300 µm distance immediately around the nerve, and Zone 2 (Z2), corresponding to a distance of 900 µm from the nerve (Figure 1B). Then, cell density was calculated by counting cell nuclei in a controlled, standard area of 0.06 mm^2^ and 0.13 mm^2^ for the Z1 and Z2 zones of the stromal compartment, respectively. The number of cells per mm^2^ was obtained by dividing the number of cells counted in HE and the area previously established, as can be observed in Supplementary Table S1. The cell proliferation rate was determined by quantifying the percentage of cells showing positive immunostaining signal for PCNA in each of these regions at both time points.

Nonetheless, to evaluate the number of CK5-negative cells and S100 and VIM-positive cells from nerve-associated cells at 7 days of time culture, we first selected the appropriate color signal using the threshold function of ImageJ. Once the positive signal was isolated, the multipoint tool was used to count the number of cells in the previously determined areas. Whereas CK5-negative values were represented per mm^2^, S100 and VIM staining results were shown as a percentage of positive cells.

Averages and standard deviations were calculated for each sample and each analysis variable. Comparisons were then performed using Mann–Whitney statistical tests [54], as most distributions were demonstrated not to accomplish parametrical standards. All statistical analyses were carried out using the Real Statistics Resource Pack software (Release 7.2) (Dr. Charles Zaiontz, Purdue University, West Lafayette, IN, USA), available at (https://www.real-statistics.com/), accessed on 3 November 2023. Statistical *p* values were corrected for all comparisons and considered statistically significant below 0.05.

## Figures and Tables

**Table 1:** Antibodies and reagents used for immunohistochemical analyses.

Antibody/Reagent	Dilution	Pretreatment	Reference
Mouse anti-human PCNA (Clone PC10)	Ready to use	EDTA, pH = 8, 95 °C for 25 min	Master Diagnostica, Granada, Spain (ref. MAD-000903QD)
Rabbit anti-cytokeratin 5	Ready to use	Citrate buffer pH = 6 95 °C for 25 min	Master Diagnostica, Granada, Spain (MAD-000491QD)
Rabbit polyclonal anti-S100 antibody (Z0311)	1: 400	Citrate buffer pH = 6 30 min at 95 °C	DakoCytomation, Glostrup, Denmark (ref. Z0311)
Mouse anti-vimentin monoclonal clone V9	1:200	Citrate buffer pH = 6 25 min at 95 °C	Merck. St. Louis, MO, USA (ref. V6389)
ImmPRESS^®^ HRP Anti-Mouse IgG (Peroxidase)	Ready to use	-	Vector Laboratories. Burlingame, USA (ref. MP-7402)
ImmPRESS^®^ HRP Anti-Rabbit IgG (Peroxidase)	Ready to use	-	Vector Laboratories. Burlingame, USA (ref. MP-7401)
Chromogen: Diaminobenzidine ready to use kit	-	-	Vector Laboratories. Burlingame, USA (ref. SK-4100)
Counterstaining: Harris Hematoxylin	30 s	-	Thermo Scientific. Runcorn, UK (ref. 6765004)

## Data Availability

The raw data supporting the conclusions of this article will be made available by the authors on request. Requests to access the datasets should be directed to the corresponding authors (ogarcia@ugr.es) or (vcarriel@ugr.es).

## Supplementary Materials

The following supporting information can be downloaded at: https://www.mdpi.com/article/10.3390/gels10040252/s1. Table S1: Group, time and zone-dependent statistical *p* values of the comparisons of different time points and of the control group (CTR), pre-invasive (Pre-Inv) and invasive (Inv) neural invasion (NI) model for each variable using the Mann–Whitney test. Statistically significant *p* values below 0.05 are labeled in red and considered statistically significant values.
